# Effects of Formulation Excipients on Skin Barrier Function in Creams Used in Pediatric Care

**DOI:** 10.3390/pharmaceutics12080729

**Published:** 2020-08-04

**Authors:** Anita Kovács, Dóra Péter-Héderi, Katalin Perei, Mária Budai-Szűcs, Attila Léber, Attila Gácsi, Erzsébet Csányi, Szilvia Berkó

**Affiliations:** 1 Institute of Pharmaceutical Technology and Regulatory Affairs, University of Szeged, Eötvös u. 6, 6720 Szeged, Hungary; anita.kovacs@pharm.u-szeged.hu (A.K.); peter-hederi.dora@med.u-szeged.hu (D.P.-H.); maria.szucs@pharm.u-szeged.hu (M.B.-S.); leber.attila@pharm.u-szeged.hu (A.L.); gacsi.attila@pharm.u-szeged.hu (A.G.); csanyi@pharm.u-szeged.hu (E.C.); 2 Department of Biotechnology, University of Szeged, Közép fasor 52, 6726 Szeged, Hungary; perei@brc.hu

**Keywords:** pediatric care, o/w cream, excipients, formulation, microbiological stability, skin barrier, skin hydration

## Abstract

Semisolid dosage forms are recommended for the dermal care of babies and children. If we look at the ingredients of these preparations, there are still many cases in which there are substances (occlusive agents, preservatives) that no longer meet certain requirements of the modern age, so it is timely to replace them with other substances. The aim of this work was to formulate a science-based formulation with new components that keep or improve its moisturizing properties, rheological parameters, and microbiological stability. Occlusive oils, like white petrolatum and liquid paraffin and the preservative parabens are traditional ingredients in oil in water creams, were replaced with white beeswax, sunflower oil, and phenoxyethanol, respectively. Cocoa butter, urea, and glycerol were added to improve long-lasting hydration and support the barrier function of the reformulated creams. The rheological properties of the formulations were determined. The effects of the preparations on skin hydration and on the barrier function of the skin were tested. Furthermore, microbiological stability was investigated. The result of the reformulation was an o/w cream that provided a good longer-lasting hydration effect; supported the barrier function of the baby skin without occlusion; and had adequate consistency, easy spreading, a pleasant skin feeling, proper pH, and good microbiological stability.

## 1. Introduction

The formulation of topical dosage forms like semisolids benefits from functional ingredients that improve product performance and ensure delivery. Semisolid systems can be found in both cosmetic and pharmacy practices. The ingredients of these formulations can affect the functional properties of the preparations; therefore, there is a need to select the proper excipients. However, the number of ingredients that can be used for formulations is much lower in pharmaceutical practice. There are a number of cosmetic and pharmaceutical products that are recommended for nursing infants and children, and also to adults for skin care. If we look at the ingredients of these creams, in many cases, there are still substances (occlusives, preservatives) that can clog skin pores, thereby irritating the skin or causing allergic reactions in susceptible individuals, especially in infants and young children [1,2]. Therefore, it is very important to apply proper ingredients in baby care preparations.

The structure and function of the baby’s skin are significantly different from the skin of adults. The infant’s skin is made up of three layers, but each layer is thinner, so it is more vulnerable. The thickness of the newborn’s skin is about 40–60% of adult skin [3]. It takes 8 to 10 weeks after birth for the stratum corneum to reach full maturity, during which the skin is unprotected, so it is more exposed to possible nosocomial infections [4]. The hydration state of the skin in newborns is low, and during the next 2–4 weeks, it increases significantly due to the onset of sweat glands. The low water content is assumed to be a consequence of adaptation to the new dry environment and the lack of a lipid matrix, as the enzymes responsible for the formation of the intercellular lipid matrix have insufficient activity in the less acidic skin surface of neonates [5]. Normal skin flora need time to develop after birth. At birth, the pH of the skin is greater than 6 (pH = 6.6–7.5 depending on the site of measurement) due to the effect of alkaline (pH = 7.4) amniotic fluid, the lack of natural bacterial flora and amino acids, and the inability of the skin enzyme system. After birth, the pH of the skin constantly decreases and after about 4 days it reaches pH = 5, where the acid barrier is created, which is ideal for skin flora and provides adequate protection. It is worth noting, however, that this is not true for the entire skin surface: For example, the closed and moisture-exposed diaper area may have a considerably higher pH [4].

Healthy mature newborns have a good barrier function at birth, while premature babies have 10 times higher transepidermal water loss (TEWL) than adults [6]. The highest TEWL values were measured at 4 h after birth, which can be explained by adaptation to the dry environment. Then, the TEWL value is set to 10 g/m^2^/h, which is a typical number of healthy adults. High TEWL may indicate skin irritation; for example, in diaper-affected areas, the value can be extremely high [7].

Some components are not to be used for children under three years of age, according to the Cosmetic Directive [8]. However, it is expected not only for baby skin but also for adult skin with many dermatological problems to develop such a formulation that does not contain any excipients that clog the skin pores, thereby causing skin irritation (e.g., occlusive agents). The two most commonly used occlusive agents are liquid paraffin and white petrolatum. They create a hydrophobic layer on the skin and physically block transepidermal water loss. It is advantageous in the case of certain diseases, for example, atopic dermatitis, but it is known that these components provide a greasy skin feeling, and cause the skin to develop acne due to their occlusive effect [9,10,11,12].

Another cause of skin irritation may be the type of preservative used to provide the microbiological stability of the preparation. Contamination of semisolid dermal formulations with various microorganisms, whether cosmetics or pharmaceuticals, results in stability problems in the product and, in the presence of microbiological contamination, also poses a risk to the health of the consumer. Therefore, it is necessary to select a suitable preservative in the preparation, which prevents the growth of microorganisms during production, storage, and use [13].

The most widely used preservatives include the salts and esters of 4-hydroxybenzoic acid, commonly called “parabens”. Recently, many changes have been made to cosmetic products as regards the regulations on these preservatives. The “iso” derivatives have been banned and the maximum permitted levels of “propyl” and “butyl” derivatives have been reduced [14]. Methyl parahydroxybenzoate is used as a preservative in many pharmaceutical products according to the 2019 Directive (Annex to the European Commission guideline on ‘Excipients in the labelling and package leaflet of medicinal products for human use’) [15]. The directive contains this substance as it may cause an allergic reaction [16] when applied on the skin. Based on this, it is justified to use a less risky preservative.

The aim of the experimental work was to find a possible way to replace occlusive components and preservative parabens, which can be found in many creams and are generally official in pharmacopoeias and guides [17,18,19] and to formulate a fragrance-free oil in water (o/w) cream that reduces possible skin irritation but does not affect or even improves the moisturizing properties and the rheological parameters of the cream, thereby further assisting the skin penetration of the incorporated active ingredients, and has adequate pH and microbiological stability.

## 2. Materials and Methods 

### 2.1. Materials

The components of the o/w cream (polysorbate 60, liquid paraffin, cetostearyl alcohol, white petrolatum, methyl parahydroxybenzoate, ethanol 96%, sunflower oil, white beeswax, cocoa butter, glycerol, urea) were purchased from Hungaropharma Ltd. (Budapest, Hungary). The preservative component phenoxyethanol was obtained from Dr Streatmans GmbH (Biesterfeld, Budapest, Hungary).

### 2.2. Plan of Reformulation

In most cases, skin irritation is caused by the preservatives in the semisolid formulations, so in the first phase of reformulation, methyl parahydroxybenzoate was replaced with phenoxyethanol as an up-to-date preservative [15,16]. It is water miscible, has broad antimicrobial performance, and has been widely used in the cosmetics industry in the last few years. The microbiological efficacy of the new preservative was tested using the “challenge test”, which is well-known in the cosmetics industry, and with the method according to the Pharmacopoeia for the microbiological purity of preparations intended for the skin [20].

In the second phase of reformulation, the petroleum derivatives (liquid paraffin and white petrolatum) were replaced with natural oil and wax (sunflower oil and white beeswax).

Sunflower oil is characterized by its low occlusive chemical components, including essential fatty acids, proteins, vitamins (B1, B2, and vitamin E with antioxidant properties), minerals, and trace elements [21]. White beeswax has a number of advantages: It forms a film coating on the skin, which does not clog the pores, does not inhibit skin breathing as opposed to mineral films, improves skin regenerative capacity, has bactericidal and calming properties, and prevents inflammation [22].

**Figure 1:**
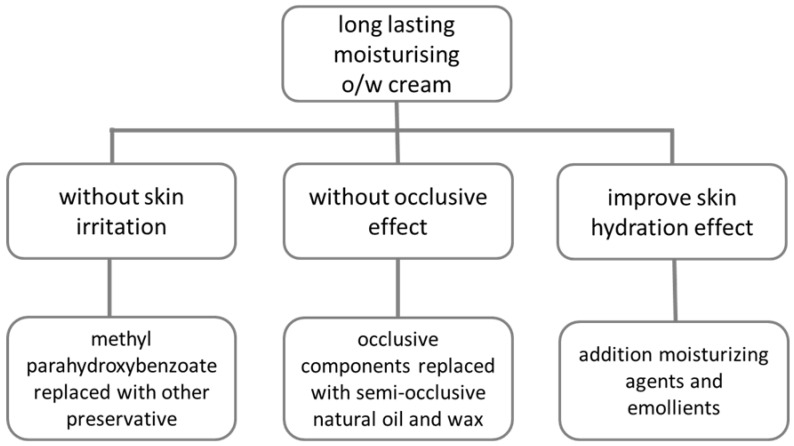
Plan of the reformulation.

The third phase of the reformulation aimed to improve skin hydration. Skin hydration can be improved by two different mechanisms of action. Emollients plasticize and soften the skin, by filling in the void spaces between the corneocytes. Emollients can also provide protection and lubrication on the skin surface, thereby reducing transepidermal water loss. Alternatively, it is possible to add substances with a water-binding property that penetrate into the deeper layers of the stratum corneum and bind water, thus increasing hydration. Additional compositions were supplemented with cocoa butter, urea, and glycerol in various combinations to improve the hydration effect (Figure 1).

### 2.3. Preparation of the Samples

The preparation of o/w creams was made with the following procedure: The oil phase (phase A) and water phase (phase B) were measured separately. Both phases were heated up to 70 °C. The water phase was added to the oil phase under stirring (Stirrer DLH, VELP Scientifica, Long Island, NY, USA, 1000 rpm). The preservative component (phase C) was added at 30 °C, and the preparation was stirred until cold.

### 2.4. Methods

#### 2.4.1. Microbiological Quality of Non-Sterile Pharmaceutical and Cosmetic Preparations for Dermal Use

There are many guidelines for testing the microbiological stability of semisolid preparations, such as the European Pharmacopoeia, the United States Pharmacopeia (USP), the Association of Southeast Asian Nations (ASEAN)Cosmetics Association, own protocols (e.g., Schülke Koko test), and the International Organization for Standardization (ISO) 11930 standard [23,24]. 

Two of these methods were used to study the formulation containing the original methyl parahydroxybenzoate and the novel phenoxyethanol. One method is the pharmacopoeial method described by the European Pharmacopoeia [20], the other is the so-called “challenge test” used in the cosmetics industry [24]. When the two methods are compared, there are some differences; the pharmacopoeia method tests the efficacy of antimicrobial preservation concerning dermal semisolid formulations and lasts for 4 weeks, while the microbiological “challenge test” for cosmetics takes 6 weeks. In the case of the pharmacopoeia method, there are no weekly inoculations with the inoculums, only one seeding at the beginning of the test with 4 different microorganism strains separately, and weekly sampling is conducted to monitor the changes in the number of viable microorganisms. In the case of the “challenge test”, there are weekly inoculations with a mixture of the inoculums of the 4 different microorganism strains and there is weekly monitoring.

The test method is as follows:

Test microorganisms:

1. *Pseudomonas aeruginosa*;
2. *Staphylococcus aureus*;
3. *Aspergillus brasiliensis*; and
4. *Candida albicans*.

Preparation of inoculum

Preparatory to the test, we inoculated the surface of casein soya bean digest agar for bacteria or sabouraud-dextrose agar without the addition of antibiotics for fungi with the recently grown stock culture of each of the specified microorganisms. The fresh stock cultures of the given microorganisms were used. The bacterial cultures were incubated at 30–35 °C for 18–24 h, the culture of *Candida albicans* at 20–25 °C for 48 h, and the culture of *Aspergillus brasiliensis* at 20–25 °C for 1 week or until good sporulation was obtained. Subcultures may be needed after revival before the microorganism is in its optimal state, but it is recommended that their number is kept to a minimum [20].

From day 0, for 6 weeks, samples were collected at the zero-point hour and weekly from two different points of the formulations in order to enumerate the viable microorganisms. Samples were not spread directly onto the agar plates but washed twice with a normal saline solution to remove the remaining antimicrobial agent in the creams. During the washing phase, two sets of centrifugation were used (13,000 RPM for 5 min). The suspended microorganisms were totally spread onto the surface of suitable agar plates.

Two different experiments were carried out. In experiment one, only one inoculation was made with the 4 strains separately at the beginning. It was followed by a weekly sample collection and viable microorganism enumeration for 4 weeks (pharmacopoeia method). In the other experiment, samples were inoculated with the mixed inoculums of the test microorganisms, inoculations were repeated weekly for 6 weeks, and the change in the number of viable microorganisms was monitored for 6 weeks (“challenge test” for cosmetics).

The course of the microbiological investigation was designed and implemented on the basis of pharmacopeia standards. At the beginning of the experiment, all the samples were inoculated with a suspension of 10^5^ to 10^6^ viable microorganisms per gram of the cream, the volume of which did not exceed 1 *v/v*% of the preparations. Only fresh inoculums were used to inoculate the cream samples. The exact number of bacteria or conidia was counted with a Bürker counting chamber in a microscope before inoculation. The concentration of the applied culture with which the inoculation was made was 0.01 mL/g. During incubation, samples were held at 25 °C and protected from the light.

Investigations were carried out in two separate sets of experiments, where the microbiological stability of creams with different preservatives was compared: The original cream with methyl parahydroxybenzoate and others containing three different concentrations (0.2, 0.5, and 1.0 m/m%) of phenoxyethanol. A preservative-free original cream was used as a control. Two parallel investigations were carried out as series A and B.

#### 2.4.2. Rheological and pH Measurements

The rheological properties were studied with a Physica MCR101 rheometer (Anton Paar, Graz, Austria). The measuring device was of the parallel plate type (diameter 25 mm, gap height 0.10 mm). The flow curves were recorded over the shear rate range from 0.1 to 100 and from 100 to 0.1 1/s at 32 °C. 

The pH of the formulations was measured with a Testo 206 pH meter. The probe of the device was immersed into 3 different parts of the sample.

#### 2.4.3. Hydration and Transepidermal Water Loss Tests

A Corneometer CM 825 (Courage and Khazaka Electronic GmbH, Cologne, Germany) is a commonly used instrument worldwide to determine the level of hydration of the skin surface, mainly the stratum corneum [25,26]. The investigation is based on the measurement of the capacitance of the dielectric medium. A Tewameter TM 300 (Courage and Khazaka Electronic GmbH, Cologne, Germany) is the most generally accepted measuring device for the assessment of transepidermal water loss (TEWL) [27]. This is a very good parameter for evaluation of the barrier function of the stratum corneum. High TEWL values indicate a greater water loss and are consistent with increased damage to the barrier function of the stratum corneum, such as may occur during irritant exposure. The probe indirectly measures the density gradient of water evaporation from the skin via the two pairs of sensors inside the hollow cylinder [28,29].

During the measurements, the changes in the state of the skin were studied on the forearms of eight volunteers (with the approval of the Ethical Committee of the University of Szeged, Albert Szent-Györgyi Clinical Centre, Human Investigation Review Board, license number: 91/2008). Female volunteers were between the age of 25 and 50 years and had healthy skin. They did not use any cosmetics on their forearm for the 24 h prior to the measurement to prevent them from influencing the results. The measurement at point zero was of the untreated state of the skin. Then, 200 mg of the compositions were applied to the designated area, left for 30 min, the excess was cleansed, and corneometric and tewametric measurements were performed at 30, 90, and 150 min.

#### 2.4.4. Statistical Analysis

The results were evaluated and analyzed statistically with the two-way analysis of variance test (Bonferroni’s multiple comparison), using Prism for Windows software (GraphPad Software Inc., La Jolla, CA, USA). The data are the averages of the results of eight volunteers ± standard deviation (* *p* < 0.05, ** *p* < 0.01, and *** *p* < 0.001 versus the original cream).

## 3. Results and Discussion

The formulation that was investigated (as original cream) was a fragrance-free o/w cream generally used in the pharmaceutical and cosmetic market, recommended as a bath cream and as a body lotion for nursing infants and children (Table 1).

It can be seen that the ingredients of these creams include occlusive agents and parabens that can irritate the skin and cause allergic reactions in susceptible individuals, especially in infants and young children.

### 3.1. Reformulation Phase 1

In the reformulation Phase 1, paraben was replaced with phenoxyethanol as an up-to-date preservative and a microbiological investigation was carried out to determine if the concentration of the different preservatives incorporated into the formulations was enough to achieve the desired goal, i.e., the growth inhibition of bacteria and fungi seeded into the creams as microbiological contaminants. The basis of the experiments was whether the model bacterial and fungal strains were present in the creams at the beginning of the experiments (initial time) and after 6 weeks with weekly sample collections in the case of the “challenge test” for cosmetics. During the “challenge test”, weekly inoculations were made with the mixture of given microorganisms to demonstrate that the preservatives in the creams could maintain microbiological quality for a longer period.

The results of the microbiological investigation indicated that the bacteria and fungi used in the experiments were not present in any of the samples after one day, which means that the preservatives in question may provide the desired stability. 

The microbiological quality was maintained in all samples during the 6-week period (the antimicrobial effect was maintained throughout the 6-week experiment by all the different preservative materials). The results in Table 2 suggest that no microorganism was present in any of the samples even with weekly inoculations. In the control formulations, the model fungal and bacterial strains were present after 1 day and in the samples obtained weekly.

The outcome of the experiment suggests that the preservative mixture applied as a substitute for methyl parahydroxybenzoate in o/w formulations in concentrations of 0.5 and 1.0 m/m% may provide an adequate microbiological quality since none of the tests showed the presence of inoculated microorganisms. However, 0.2 m/m% of phenoxyethanol may not be protective against the growth of *Candida albicans* (Table 3).

According to the results, the maximum allowed concentration (1 m/m%) of phenoxyethanol in regulations on cosmetic products may be decreased to 0.5 m/m%, which provides adequate microbiological stability [8].

### 3.2. Reformulation Phase 2

In the reformulation phase 2, liquid paraffin and white petroleum were replaced with sunflower oil and white beeswax. The following four reformulated compositions were prepared (Table 4).

In composition 1, liquid paraffin was replaced with sunflower oil and white petrolatum was replaced with white beeswax in the same percentage according to the original cream. When this preparation was applied, bad spread and an unpleasant skin feeling were detected, which was attributable to high white beeswax concentrations. In compositions 2 and 3, the amount of white beeswax was gradually reduced to achieve the appropriate consistency. In composition 4, besides reducing white beeswax, the amount of sunflower oil was increased.

**Figure 2:**
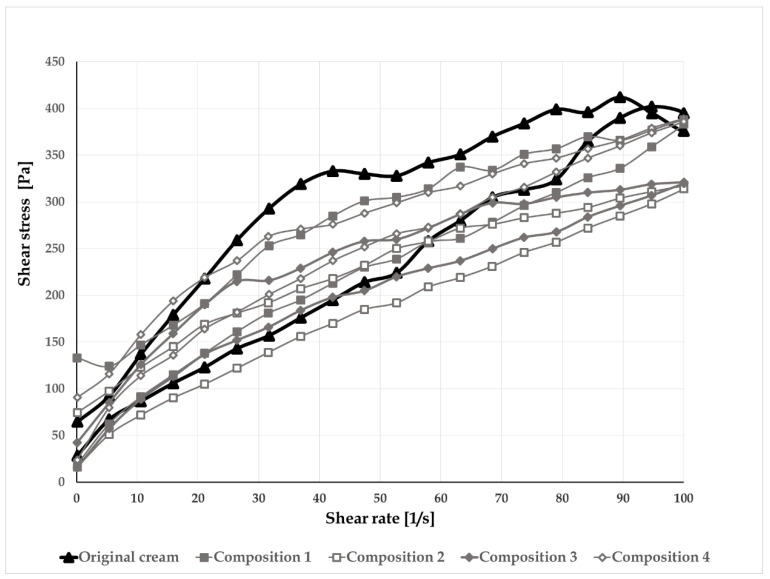
Flow curves of the preparations.

O/w creams containing mixed emulsifiers are at a minimum four-phase systems, such as crystalline/hydrophilic gel phase (bilayers of surfactant and fatty amphiphile, where the water and other hydrophilic components can be inserted resulting in interlamellar water); bulk water phase, lipophilic gel phase, and dispersed oil phase [30] The gel phases are responsible for the coherent structure of the creams. The forces acting in the coherent structure are in correlation with the viscosity of the systems. The creams show plastic flow behavior (Figure 2) with a yield stress value. A special rheological characteristic of the creams is the presence of bulge in the up curve of the flow curve. This phenomenon is typical for the organogels, where the gel network is broken down before it starts to flow. During the flow curve rotational test, usually, the structural breakdown is investigated, and the hysteresis area can mean the value for the structural breakdown. The presence of thixotropy means satisfactory spreadability on the skin and easy extraction from the tube.

On the basis of the international guideline of European Medicines Agency (EMA) [31], for the evaluation of the microstructure of the creams, rheological data from the flow curves were collected, such as the viscosity at specified shear rates (η_100_, viscosity at 100 1/s) and the thixotropic relative area (S_R_, where the sample volume is 0.2 mL) (Table 5).

Comparing the flow curves and the rheological data of the formulations, higher white beeswaxes and higher oil-containing compositions showed similar results to the original cream. However, creams containing less wax and thus more water (compositions 2 and 3) were softer in consistency, better lubricated, and more comfortable with the skin, so composition 3 was chosen for further investigation.

When evaluating the moisturizing effect, the values of untreated skin were taken to zero, and relative to this, the changes were presented as a percentage.

**Figure 3:**
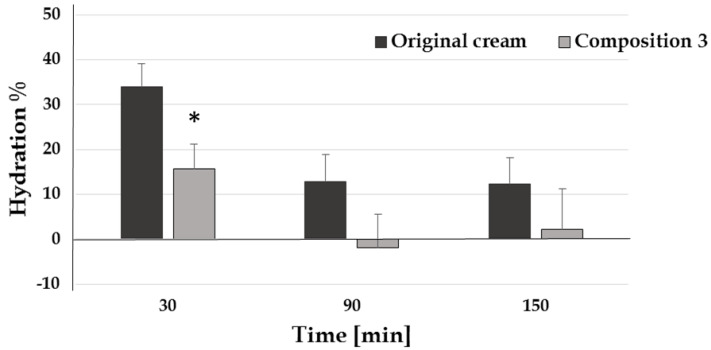
Hydration effect of the preparations (*: *p* < 0.05 vs. original cream).

During the hydration measurements, it was found that the moisturizing capacity of the reformulated formulation is below that of the original cream (Figure 3). The changing of the composition caused a lower hydration effect so further components should be added to composition 3 to reach or to improve the hydration effect of the original cream.

**Figure 4:**
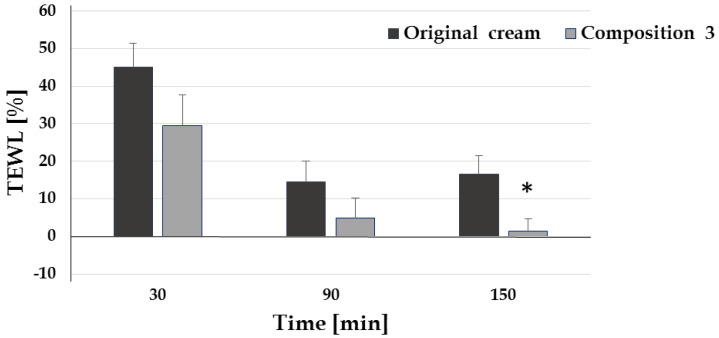
TEWL measurement of the preparations (*: *p* < 0.05 vs. original cream).

Transepidermal water loss was reduced by reformulation 3. The TEWL values predict the barrier function of the skin. White beeswax and sunflower oil, the changed components in the reformulated cream, showed less of an effect on the structure of stratum corneum lipids; therefore, composition 3 is better for the skin barrier (Figure 4).

The pH of the healthy skin is slightly acidic: pH = 5.4–5.9. The pH values of the preparations produced vary from 4.52 to 4.89 (Table 6), which are generally accepted and favorable for maintaining the acidic pH of the skin.

### 3.3. Reformulation Phase 3

In phase 3 of the reformulation, the aim was to improve skin hydration, which was achieved by two mechanisms of action. Using further emollients (cocoa butter), which form a thin layer on the skin surface and reduce TEWL, or adding substances (urea, glycerol) with a water-binding property that penetrate into the deeper layers of the skin, bind the water, and thereby increase hydration. Composition 3 was supplemented with new components in various combinations, according to Table 7.

**Figure 5:**
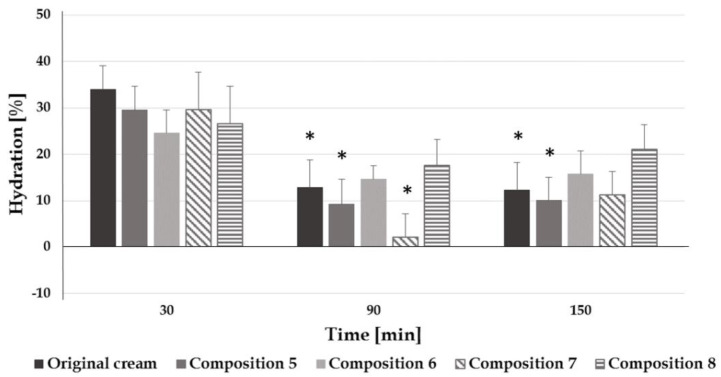
Hydration effect of the preparations (*: *p* < 0.05 vs. 30 min).

When assessing the results, the hydration of the stratum corneum improved after 30 min, and there was no significant difference in the moisturizing effect of each formulation. However, in the original cream and in compositions 5, 6, and 7, it can be seen that hydration decreased significantly after 90 and 150 min, which means that the preparations hydrate the skin, but a significant part of the water leaves it through transepidermal water loss, thus the preparations have no permanent moisturizing effect. However, in the case of composition 8, which contains a combination of cocoa butter and glycerol, the hydration level was permanent throughout the test period (no significant decrease can be observed), which predicts a longer-lasting moisturizing effect of this composition (Figure 5).

**Figure 6:**
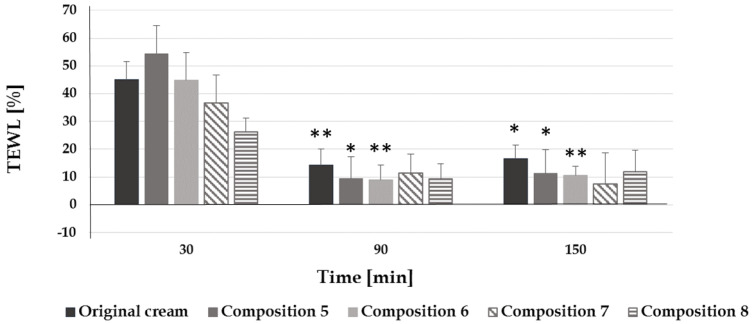
Effect of the preparations on TEWL (*: *p* < 0.05 vs. 30 min, **: *p* < 0.01 vs. 30 min).

Higher TEWL values after 30 min support the hydration results, since there is no permanent hydration if the TEWL value is higher (Figure 6). If the TEWL value increases, hydration will decrease in most cases because the water content of the stratum corneum leaves through transepidermal water loss. In the formulation where the composition can decrease the TEWL, hydration will be higher for a longer period. 

**Figure 7:**
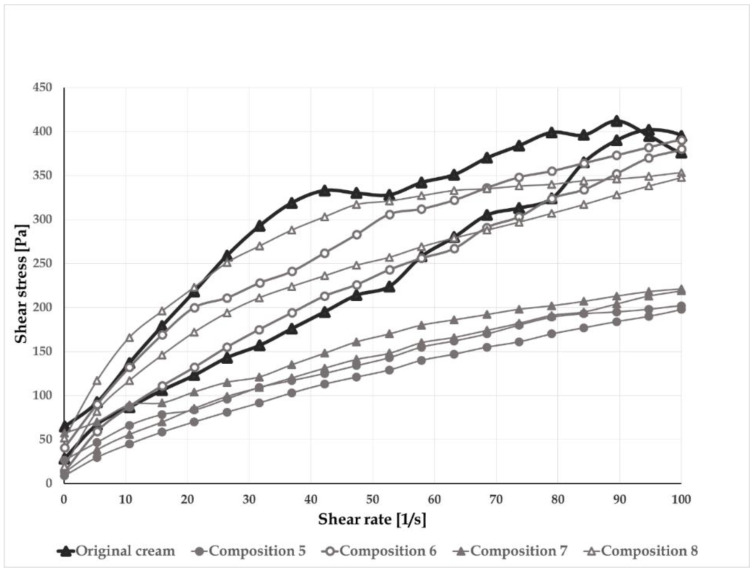
Flow curves of the preparations.

In addition to good hydration, the consistency of the preparation is also essential. In Table 8 and Figure 7, the rheological parameters of the compositions are presented. The viscosity of all modified compositions is lower than that of the original cream, but composition 6 and 8 were very similar to the original one. In conclusion, it can be said that the spreadability and skin sensation of the compositions are the same or better than those of the original formulation.

The pH of these preparations varied from pH = 4.52–5.07 (Table 9), which is favorable for maintaining the acidic pH of the skin.

**Figure 8:**
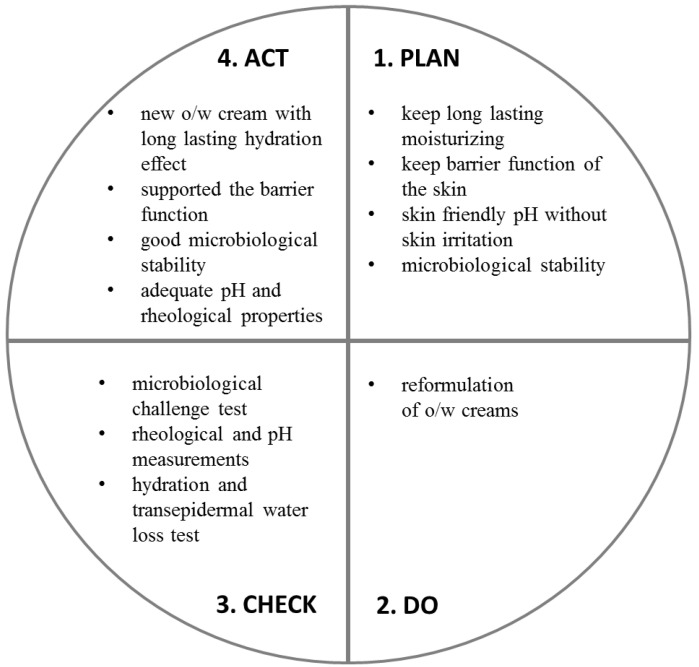
The PDCA cycle of the reformulation work.

The reformulation work was summarized using the PDCA (plan-do-check-act) cycle. The PDCA cycle is a four-step method used for the control and continual improvement of the process and product (Figure 8). 

## 4. Conclusions

There are many excipients in the pharmaceutical and cosmetic fields for the renewal of official compositions to replace obsolete components for better hydration and skin barrier function with adequate rheological and pH properties and good microbiological stability. The present study described the impact of excipients on the quality of semisolid formulations and their applicability for dermal use in pediatric care. 

The aim of the study was to replace occlusive components with semi-occlusive ones and the old paraben-type preservative with a newer one in an official o/w cream. After replacing the component, it was expected to check the features of the cream (i.e., hydration, TEWL, microbiological stability, rheology, and pH) and change the composition if the parameters were not appropriate. The result of the reformulation was an o/w cream, which provided a good longer-lasting hydration effect; supported the barrier function of the baby skin without occlusion; and had adequate consistency, easy spreading, a pleasant skin feeling, proper pH, and good microbiological stability for pediatric care use.

## Figures and Tables

**Table 1:** Composition of the original cream [19].

Component	Concentration [*w*/*w*%]	Function
**Phase A**		
Polysorbate 60	4	nonionic o/w emulsifier
Liquid paraffin	4	oil phase and consistency softener
Cetostearyl alcohol	12	nonionic w/o emulsifier and consistency-increasing agent
White petrolatum	20	base of the cream
**Phase B**		
Purified water	58	water phase of the cream
**Phase C**		
Methyl parahydroxybenzoate	0.2	preservative
Ethanol 96%	1.8	solvent of preservative

**Table 2:** Results of microbiological investigations in the case of the control cream with no preservative incorporated.

	Not Infected	*Pseudomonas aeruginosa*	*Staphylococcus aureus*	*Aspergillus brasiliensis*	*Candida albicans*	Mixed 25–25% Reinfected
**Control A**	Initial time: 0	Initial time: 2 × 10^5^	Initial time: 3 × 10^5^	Initial time: 1 × 10^5^	Initial time: 1 × 10^5^	Initial time: 1.75 × 10^5^
	1 day–6 weeks: 0	1 day–6 weeks: 1 × 10^5^	1 day: 2 × 10^5^	1 day: 1 × 10^5^	1 day: 7 × 10^4^	1 day: 1.2 × 10^5^
			1–6 weeks: 0	1–6 weeks: 2 × 10^1^	1–6 weeks: 6 × 10^4^	1–6 weeks: 3 × 10^3^
**Control B**	Initial time: 0	Initial time: 2 × 10^5^	Initial time: 3 × 10^5^	Initial time: 1 × 10^5^	Initial time: 1 × 10^5^	Initial time: 1.75 × 10^5^
	1 day–6 weeks: 0	1 day–6 weeks: 1 × 10^5^	1 day: 2 × 10^5^	1 day: 1 × 10^5^	1 day: 7 × 10^4^	1 day: 1.2 × 10^5^
			1–6 weeks: 0	1–6 weeks: 2 × 10^1^	1–6 weeks: 6 × 10^4^	1–6 weeks: 3 × 10^3^

**Table 3:** Results of microbiological investigations in the case of formulations containing a preservative excipient (Sample 1: original cream containing 0.2 m/m% methyl parahydroxybenzoate, Sample 2: original cream containing phenoxyethanol 0.2 m/m%, Sample 3: original cream containing 0.5 m/m% phenoxyethanol, Sample 4: original cream containing 1.0 m/m% phenoxyethanol).

	Not Infected	*Pseudomonas aeruginosa*	*Staphylococcus aureus*	*Aspergillus brasiliensis*	*Candida albicans*	Mixed 25–25% Reinfected
**Sample1 A**	Initial time: 0	Initial time: 2 × 10^5^	Initial time: 3 × 10^5^	Initial time: 1 × 10^5^	Initial time: 1 × 10^5^	Initial time: 2–3 × 10^5^
	1 day–6 weeks: 0	1 day–6 weeks: 0	1 day–6 weeks: 0	1 day–6 weeks: 0	1 day–6 weeks: 0	1 day–6 weeks: 0
**Sample1 B**	Initial time: 0	Initial time: 2 × 10^5^	Initial time: 3 × 10^5^	Initial time: 1 × 10^5^	Initial time: 1 × 10^5^	Initial time: 2–3 × 10^5^
	1 day–6 weeks: 0	1 day–6 weeks: 0	1 day–6 weeks: 0	1 day–6 weeks: 0	1 day–6 weeks: 0	1 day–6 weeks: 0
**Sample2 A**	Initial time: 0	Initial time: 2 × 10^5^	Initial time: 3 × 10^5^	Initial time: 1 × 10^5^	Initial time: 1 × 10^5^	Initial time: 1.75 × 10^5^
	1 day–6 weeks: 0	1 day–6 weeks: 0	1 day–6 weeks: 0	1 day–6 weeks: 0	**1 day:** **4 × 10^4^**	**1 day:** **1 × 10^5^ (Candida!)**
					**1–6 weeks:** **2 × 10^4^**	**1–6 weeks:** **1 × 10^3^(Candida!)**
**Sample2 B**	Initial time: 0	Initial time: 2 × 10^5^	Initial time: 3 × 10^5^	Initial time: 1 × 10^5^	Initial time: 1 × 10^5^	Initial time: 1.75 × 10^5^
	1 day–6 weeks: 0	1 day–6 weeks: 0	1 day–6 weeks: 0	1 day–6 weeks: 0	**1 day:** **4 × 10^4^**	**1 day:** **1 × 10^5^ (Candida!)**
					**1–6 weeks:** **2 × 10^4^**	**1–6 weeks:** **1 × 10^3^(Candida!)**
**Sample3 A**	Initial time: 0	Initial time: 2 × 10^5^	Initial time: 3 × 10^5^	Initial time: 1 × 10^5^	Initial time: 1 × 10^5^	Initial time: 2–3 × 10^5^
	1 day–6 weeks: 0	1 day–6 weeks: 0	1 day–6 weeks: 0	1 day–6 weeks: 0	1 day–6 weeks: 0	1 day–6 weeks: 0
**Sample3 B**	Initial time: 0	Initial time: 2 × 10^5^	Initial time: 3 × 10^5^	Initial time: 1 × 10^5^	Initial time: 1 × 10^5^	Initial time: 2–3 × 10^5^
	1 day–6 weeks: 0	1 day–6 weeks: 0	1 day–6 weeks: 0	1 day–6 weeks: 0	1 day–6 weeks: 0	1 day–6 weeks: 0
**Sample4 A**	Initial time: 0	Initial time: 2 × 10^5^	Initial time: 3 × 10^5^	Initial time: 1 × 10^5^	Initial time: 1 × 10^5^	Initial time: 2–3 × 10^5^
	1 day–6 weeks: 0	1 day–6 weeks: 0	1 day–6 weeks: 0	1 day–6 weeks: 0	1 day–6 weeks: 0	1 day–6 weeks: 0
**Sample4 B**	Initial time: 0	Initial time: 2 × 10^5^	Initial time: 3 × 10^5^	Initial time: 1 × 10^5^	Initial time: 1 × 10^5^	Initial time: 2–3 × 10^5^
	1 day–6 weeks: 0	1 day–6 weeks: 0	1 day–6 weeks: 0	1 day–6 weeks: 0	1 day–6 weeks: 0	1 day–6 weeks: 0

**Table 4:** Components of the reformulated compositions.

Components	Original Cream [*w*/*w* %]	Composition 1 [*w*/*w* %]	Composition 2 [*w*/*w* %]	Composition 3 [*w*/*w* %]	Composition 4 [*w*/*w* %]
**Phase A**					
Polysorbate 60	4	4	4	4	4
Liquid paraffin	4	-	-	-	-
Cetostearyl alcohol	12	12	12	12	12
White petrolatum	20	-	-	-	-
Sunflower oil	-	4	4	4	8
White beeswax	-	20	15	10	5
**Phase B**					
Purified water	up to 100	up to 100	up to 100	up to 100	up to 100
**Phase C**					
Methyl parahydroxy-benzoate	0.2	-	-	-	-
Ethanol 96%	1.8	-	-	-	-
Phenoxyethanol	-	0.5	0.5	0.5	0.5

**Table 5:** Rheological data (η100 and S_R_) of the original and the modified creams.

Rheological Data	Original Cream	Composition 1	Composition 2	Composition 3	Composition 4
η_100_ (Pa *s)	4.07 ± 0.31	3.38 ± 0.50	2.84 ± 0.27	3.26 ± 0.08	3.98 ± 0.15
S_R_ Pa s^−s^ mL^−1^	39,639 ± 4631	21,028 ± 6683	21,973 ± 2593	26,504 ± 6561	18,341 ± 4618

**Table 6:** pH values of the preparations.

Original Cream	Composition 1	Composition 2	Composition 3	Composition 4
4.52	4.81	4.76	4.89	4.88

**Table 7:** Components of the reformulated compositions.

Components	Original Cream [*w*/*w*%]	Composition 5 [*w*/*w*%]	Composition 6 [*w*/*w*%]	Composition 7 [*w*/*w*%]	Composition 8 [*w*/*w*%]
**Phase A**					
Polysorbate 60	4	4	4	4	4
Liquid paraffin	4	-	-	-	-
Cetostearyl alcohol	12	6	12	6	12
White petrolatum	20	-	-	-	-
Sunflower oil	-	4	4	4	4
White beeswax	-	10	10	10	8
Cocoa butter	-	6	-	6	8
**Phase B**					
Urea	-	-	1	1	-
Glycerol (85%)	-	-	-	-	5
Purified water	up to 100	up to 100	up to 100	up to 100	up to 100
**Phase C**					
Methyl parahydroxy-benzoate	0.2	-	-	-	-
Ethanol 96%	1.8	-	-	-	-
Phenoxyethanol	-	0.5	0.5	0.5	0.5

**Table 8:** Rheological data (η100 and S_R_) of the original and the modified creams.

Rheological Data	Original Cream	Composition 5	Composition 6	Composition 7	Composition 8
η_100_ (Pa*s)	4.07 ± 0.31	1.94 ± 0.12	3.54 ± 0.38	2.11 ± 0.14	3.50 ± 0.05
S_R_ Pa s^−s^ mL^−1^	39,639 ± 4631	11,696 ± 643	24,218 ± 2811	6051 ± 28	15,698 ± 1104

**Table 9:** pH values of the preparations.

Original Cream	Composition 5	Composition 6	Composition 7	Composition 8
4.52	4.65	5.07	5.02	4.60
